# Risk of stroke and myocardial infarction after influenza-like illness in New York State

**DOI:** 10.1186/s12889-021-10916-4

**Published:** 2021-05-05

**Authors:** Erin R. Kulick, Trevor Alvord, Michelle Canning, Mitchell S. V. Elkind, Bernard P. Chang, Amelia K. Boehme

**Affiliations:** 1 Department of Epidemiology and Biostatistics, Temple University College of Public Health, 1301 Cecil B Moore Avenue, Ritter Annex 904, Philadelphia, PA 19122 USA; 2 Department of Epidemiology, Brown University, Providence, RI USA; 3 Department of Neurology, Vagelos College of Physicians and Surgeons, Columbia University, New York City, NY USA; 4 Department of Epidemiology, Mailman School of Public Health, Columbia University, New York, NY USA; 5 Department of Emergency Medicine, Vagelos College of Physicians and Surgeons, Columbia University, New York City, NY USA

**Keywords:** Cardiovascular diseases, Influenza, Myocardial infarction, Stroke, Case-crossover, Health disparities

## Abstract

**Background:**

Influenza may be associated with increased stroke and myocardial infarction (MI) risk. We hypothesized that risk of stroke and MI after influenza-like illness (ILI) would be higher in patients in New York State. We additionally assessed whether this relationship differed across a series of sociodemographic factors.

**Methods:**

A case-crossover analysis of the 2012–2014 New York Statewide Planning and Research Cooperative System (SPARCS) was used to estimate odds of ischemic stroke and MI after ILI. Each patient’s case window (the time period preceding event) was compared to their control windows (same dates from the previous 2 years) in conditional logistic regression models used to estimate odds ratios and 95% confidence intervals (OR, 95% CI). We varied the case windows from 15 to 365 days preceding event as compared to control windows constructed using the same dates from the previous 2 years. Analyses were stratified by sex, race, and urban-rural status based on residential zip code.

**Results:**

A total of 33,742 patients were identified as having ischemic stroke and 53,094 had MI. ILI events in the 15 days prior were associated with a 39% increase in odds of ischemic stroke (95% CI 1.09–1.77), increasing to an almost 70% increase in odds when looking at ILI events over the last year (95% CI 1.56, 1.83). In contrast, the effect of ILI hospitalization on MI was strongest in the 15 days prior (OR = 1.24, 95% CI 1.06–1.44). The risk of ischemic stroke after ILI was higher among individuals living in rural areas in the 90 days prior to stroke and among men in the year prior to event. In contrast, the association between ILI and MI varied only across race with whites having significantly higher ILI associated MI.

**Conclusion:**

This study highlights risk period differences for acute cardiovascular events after ILI, indicating possible differences in mechanism behind the risk of stroke after ILI compared to the risk of MI. High risk populations for stroke after ILI include men and people living in rural areas, while whites are at high risk for MI after ILI. Future studies are needed to identify ways to mitigate these risks.

## Background

The morbidity and mortality associated with cardiovascular disease (CVD) is high. Heart Disease is the number one cause of death in the US, with approximately 735,000 myocardial infarction (MI) events per year [1]. While the mortality from stroke has decreased, it is the fifth leading cause of death in the US and remains the leading cause of long-term adult disability with 800,000 stroke events annually [1, 2]. Despite decreases in mortality, the morbidity associated with these diseases remains high, with a cost burden of approximately $17.5 billion per year for direct stroke costs and $11.3 billion for direct MI costs [1–3]. The reduction of risk factors for stroke and MI has become a high priority, with policy efforts implementing risk reduction efforts [3]. Traditional cardiovascular risk factors such as diabetes, smoking, high blood pressure, and sedentary behavior comprise only 80% of the risk associated with incident events. In addition, these factors are largely associated with long-term risk and do not account for short-term risk or a triggering event, i.e., the reason why an event occurs at a particular point in time, despite risk factors being present for years [4, 5]. Therefore, with a substantial proportion of risk unexplained, identification of novel short term risk factors is of great importance.

Common infections, such as respiratory tract infections, have been identified as both an acute, short term trigger for stroke and MI and as a long-term chronic risk factor [6–15] . Respiratory tract infections are the most common cause of infection in adults, and “Influenza-like illness” (ILI) accounts for the majority of these infections [16]. Previous studies have shown significant associations between ILI and acute cardiovascular events, however no study has addressed these relationships in a large scale, generalizable dataset that allows for exploration of geographical, urban/rural, and racial-ethnic differences that could further influence these relationships. We hypothesized that ILI is associated with increased risk of incident stroke and MI in New York State. We additionally assessed whether this relationship differed by a series of sociodemographic factors and hypothesized that the risk of ILI associated event would be higher for men, minority populations, and those living in an urban environment.

## Methods

### Study design

We conducted a case-crossover analysis using 2012–2014 data from the New York State Department of Health Statewide Planning and Research Cooperative System (SPARCS) dataset, a comprehensive data reporting system that collects information on hospital admissions and emergency department (ED) visits within the state of New York. The SPARCS dataset collects information on approximately 98% of all hospitalizations in non-federal acute care facilities regardless of insurance status. Information on patient characteristics, diagnoses, treatments, services and charges is collected for each hospitalization or ED visit. Data elements include demographic information such as age, sex, race, and residential address. A unique patient ID (‘*upid’)* is assigned to each person to allow for tracking hospitalizations and ED visits over time. Diagnoses are established using International Classification of Disease (ICD-9) codes. The study populations contained all patients older than 18 years of age hospitalized for ischemic stroke or MI during 2014 in any of the non-federal acute care hospitals in New York State.

We employed a case-crossover design where each case served as his or her own control to investigate the association between acute cardiovascular events, specifically ischemic stroke and MI occurring in 2014, and preceding ILI events from 2012 to 2014. The case-crossover design is often used to study acute events, such as stroke or MI, triggered by an exposure that increases the risk for having an event transiently [17]. In this design, data from a short risk period prior to the event (case period) is compared to another time-period (control period) in the same individual. In addition, the use of a case-crossover design removes confounding by characteristics that do not vary over time and limits confounding by seasonality and long-term trends [17, 18]. This strength is particularly important in a study of administratie 1ve health records that do not have detailed data on potential risk factors that may confound any association. Each patient’s case window (the time period preceding event) was compared to their control windows (constructed using the same dates from the previous 2 years).

### Exposure assessment

We defined ILI using previously described ICD-9 codes defined by the Department of Defense for the purposes of identifying ILI cases for surveillance [19]. Cases of ILI were defined as having one of the ICD-9 codes listed in Table 1 present on arrival at any diagnostic position for either an ED visit or a hospitalization for ILI. If no present on arrival indicator was available, we treated the diagnoses as in-hospital ILI and was not considered an exposure event. We used both inpatient and outpatient (ED) databases to capture ILI cases that were hospitalized and ILI cases that only presented to the ED department.

**Table 1:** ICD-9 Codes used to define Influenza-Like Illness (ILI)

079.89	Viral infection NEC	466	Acute bronchitis and bronchiolitis
079.99	Viral infection NOS	466.0	Acute bronchitis
460	Acute nasopharyngitis	466.1	Acute bronchiolitis
462	Acute pharyngitis	466.19	Acute bronchiolitis due to other infectious organism
464	Acute laryngitis and tracheitis	478.9	Other and unspecified diseases of upper respiratory tract
464.0	Acute laryngitis	480	Viral pneumonia
464.1	Acute tracheitis	487	Influenza
464.10	Acute tracheitis w/o obstruction	487.0	Influenza with pneumonia
464.2	Acute laryngotracheitis	487.1	Influenza with other respiratory manifestation
464.20	Acute laryngotracheitis w/o obstruction	487.8	Influenza with other manifestation
465	Upper respiratory infection, multiple or unspecified sites	490	Bronchitis not specified as acute or chronic
465.0	Acute laryngopharyngitis	780.6	Fever
465.8	Upper respiratory infection of multiple sites	784.1	Throat pain
465.9	Upper respiratory infection of unspecified sites	786.2	Cough

Adapted from Eick-Cost AA, Hunt DJ. 2015 *Assessment of ICD-9-based case definitions for influenza-like illness surveillance*. MSMR

**Fig. 1:**
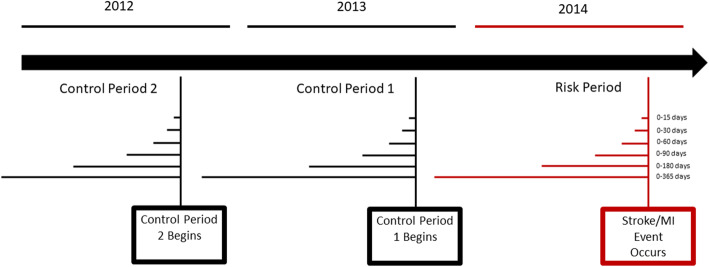
Case-Crossover Study Design. Describes the case-crossover study design used in this analysis and compares the relationship between each patient’s case window (the time -period preceding the cardiovascular event) to their control windows (constructed using the same calendar dates from the previous two years)

We defined an exposure event as both inpatient and outpatient admission for ILI in varying time intervals (0–15, 0–30, 0–60, 0–90, 0–180, 0–365 days) prior to first stroke or MI event (defined as the case periods) and identical time intervals 1 and 2 years before the event (defined as the control periods), such that the calendar time of the year remained constant across both case and control periods (Fig. 1).

### Outcome assessment

We defined Ischemic stroke using ICD-9 codes validated in previous studies: 433.× 1 (“x,” the fourth digit, varies to specify arterial distribution), 434 (excluding 434.× 0), or 436 at any diagnostic position. We excluded cases if “traumatic brain injury” ICD-9-CM code (800 to 804, 850 to 854) or “rehabilitation care” ICD-9-CM codes (V57) was present as the primary diagnosis [15, 20, 21]. MI was defined as ICD-9 code 410 present at any diagnostic position.

The SPARCS dataset does not provide distinguishable dates ICD-9 codes within the same admission, eliminating the ability of researchers to identify any temporal relationship of events within a singlefigur hospitalization. To guarantee all analyses were done between separate admissions for cardiovascular events and ILI, we removed all admissions for which stroke or MI and ILI occurred in the same admission from the analysis.

### Covariates

The advantage to using a case-crossover design is that the study design accounts for traditional confounders in the analyses, with each case serving as their own control. We examined whether the association between ILI and cardiovascular events differed across a series of sociodemographic characteristics available in the SPARCS dataset including geographic location, race, and sex. We classified patients as living in urban versus rural communities based on their home ZIP code using Rural-Urban Commuting Area Codes (RUCA codes) previously assigned by the United States Department of Agriculture [22]. Urban ZIP codes included metropolitan communities with at least 50,000 residents. Rural ZIP codes included communities not connected to an urban center. Race, as entered into administrative records, was categorized into white versus black patients. Sex was dichotomized into male and female based on medical records.

### Statistical analysis

We used conditional logistic regression stratified on individual ID variable *“upid”* to compute odds ratios (ORs) and 95% confidence intervals (CI) for any inpatient admission for stroke or MI within 0–15 days, 0–30 days, 0–60 days, 0–180 days, and 0–365 days after exposure. We fit separate models to estimate the associations between ILI and ischemic stroke and MI.

While this study design did not allow for the investigation of time varying confounding, it has been shown in stroke and MI that limiting the control windows to a short time period (e.g. 2 years) reduces the bias from time varying confounders. For this scenario, two control periods were randomly selected from the two previous years, matching on month and date, to avoid overlap bias [23, 24].

To test whether the association of infections with cardiovascular event odds differs by available sociodemographic characteristics (sex, geography, or race), we included two-way interaction terms between each characteristic and ILI into individual conditional logistic regression models. Interaction terms with a *p*-value < 0.10 were considered potentially statistically significant [25–27]. We then calculated stratum-specific estimates to look at differences in the relationship between ILI and cardiovascular events across rural/urban status, sex, and race.

## Results

A total of 33,742 patients were identified as having an ischemic stroke and 53,094 had a MI in 2014 and were included in the analyses. Of those patients, 2009 (6%) of ischemic strokes and 3773 (7%) of MIs had at least one episode of ILI in the 365 days prior to event. On average, people who had an ILI event prior to a stroke or MI were younger and a higher proportion were black (Table 2).

**Table 2:** Baseline characteristics of patients with and without exposure to Influenza-Like-Illness in 365-days prior to cardiovascular event

	Ischemic Stroke (*n* = 33,742)		Myocardial Infarction (*n* = 53,094)	
	Cases with exposure to ILI (*n* = 2009)	Cases without ILI (*n* = 31,733)	Cases with exposure to ILI (*n* = 3773)	Cases without ILI (*n* = 49,321)
Age (years)	65.6	72.2	66.9	71.3
Standard deviation (range)	16.4 (18–101)	14.6 (18–104)	15.3 (18–109)	14.4 (18–111)
Women, N (%)	1037 (51.6%)	16,213 (51.1%)	1714 (45.4%)	21,372 (43.3%)
Race, N (%)				
White	910 (45.6%)	19,675 (62.3%)	2064 (54.9%)	34,243 (69.7%)
Black	1099 (54.7%)	12,058 (37.7%)	1709 (45.3%)	15,078 (30.3%)
Residential Geography, N (%)				
Urban	1795 (89.4%)	28,287 (89.1%)	3214 (85.2%)	42,736 (86.7%)
Rural	214 (10.6%)	3446 (10.9%)	559 (14.8%)	6585 (13.3%)

The presence of an ILI hospitalization event within all period lengths was significantly associated with increased odds of ischemic stroke. Hospitalizations for ILI in the 15 days prior were associated with a 39% increase in odds of ischemic stroke (OR = 1.39, 95% CI 1.09–1.77), increasing to a 69% increase in odds when looking at ILI events over the last year (OR = 1.69, 95% CI 1.56, 1.83) (Table 3). In contrast, the effect of ILI hospitalization on MI was significant in the 15 days prior to the event, with a 24% increase in odds of MI (OR = 1.24, 95% CI 1.06–1.44), but decreasing slightly over the following 90 days. The strongest effect was seen when including at ILI events over the last year, increasing the odds of MI by 48% (OR = 1.48, 95% CI 1.41, 1.57) (Table 3).

**Table 3:** Cumulative odds ratios and 95% CI for ILI with risk of cardiovascular event

Hospitalization for ILI	Ischemic Stroke		Myocardial Infarction	
	OR	95% CI	OR	95% CI
0–15 days before event	1.39*	1.09, 1.77	1.24*	1.06, 1.44
0–30 days before event	1.48*	1.25, 1.76	1.07	0.96, 1.20
0–60 days before event	1.48*	1.29, 1.69	1.04	0.95, 1.14
0–90 days before event	1.59*	1.42, 1.78	1.07	0.99, 1.15
0–180 days before event	1.68*	1.53, 1.85	1.21*	1.14, 1.29
0–365 days before event	1.69*	1.56, 1.83	1.48*	1.41, 1.57

*Indicates Statistical Significance at *p* < 0.05.

We next assessed whether the association between ILI and acute cardiovascular events differed across residential geography, sex, or race. In the first 30 days prior to an ischemic stroke event, the effect of ILI on risk of ischemic stroke was statistically significantly higher among individuals living in rural areas of New York State (*p*-value for interaction = 0.10) and among men (*p*-value for interaction = 0.04). There were also differences across sex in later time periods, with the effect of ILI on ischemic stroke stronger in men across a 180-day span (*p*-value for interaction = 0.08) (Table 4).

**Table 4:** Effect Modification of Association between ILI and Ischemic stroke by demographic and sociodemographic characteristics

	Residential Geography			Sex			Race		
	Urban	Rural	***p***-value for interaction	Men	Women	***p***-value for interaction	Black	White	***p***-value for interaction
	OR (95%CI)	OR (95%CI)		OR (95%CI)	OR (95%CI)		OR (95%CI)	OR (95%CI)	
0–15 days before event	1.38 (1.08, 1.78)	1.43 (0.63, 3.22)	0.98	1.57 (1.13, 2.18)	1.22 (0.85, 1.72)	0.82	1.40 (0.88, 2.24)	1.43 (0.99, 2.04)	0.79
0–30 days before event	1.45 (1.21, 1.75)	1.65 (1.05, 2.59)	0.10*	1.82 (1.43, 2.30)	1.17 (0.91, 1.51)	0.04*	1.34 (0.95, 1.89)	1.47 (1.15, 1.89)	0.56
0–60 days before event	1.48 (1.28, 1.70)	1.51 (1.07, 2.12)	0.93	1.65 (1.37, 1.98)	1.33 (1.10, 1.60)	0.11	1.36 (1.05, 1.76)	1.49 (1.23, 1.80)	0.64
0–90 days before event	1.56 (1.38, 1.76)	1.79 (1.33, 2.41)	0.56	1.75 (1.49, 2.06)	1.45 (1.23, 1.69)	0.23	1.56 (1.25, 1.95)	1.55 (1.31, 1.82)	0.36
0–180 days before event	1.69 (1.52, 1.87)	1.63 (1.29, 2.07)	0.87	1.87 (1.63, 2.14)	1.53 (1.34, 1.74)	0.08*	1.71 (1.42, 2.05)	1.52 (1.33, 1.74)	0.50
0–365 days before event	1.71 (1.57, 1.87)	1.57 (1.29, 1.91)	0.51	1.73 (1.54, 1.95)	1.66 (1.49, 1.84)	0.54	1.64 (1.41, 1.92)	1.56 (1.401, 1.744)	0.93

*Indicates Statistical Significance at *p* < 0.05

The association between ILI and MI varied significantly across race. In all time periods, the relationship between ILI and MI was significantly stronger among white individuals as compared to black individuals (Table 5). There were no significant differences across urban/rural status or sex.

**Table 5:** Effect Modification of Association between ILI and Myocardial Infarction by demographic and sociodemographic characteristics

	Residential Geography			Sex			Race		
	Urban	Rural	***p***-value for interaction	Male	Female	***p***-value for interaction	Black	White	***p***-value for interaction
	OR (95%CI)	OR (95%CI)		OR (95%CI)	OR (95%CI)		OR (95%CI)	OR (95%CI)	
0–15 days before event	1.20 (1.01, 1.42)	1.41 (0.99, 2.01)	0.26	1.15 (0.94, 1.41)	1.35 (1.07, 1.70)	0.91	1.13 (0.78, 1.62)	1.63 (1.32, 2.01)	0.08*
0–30 days before event	1.02 (0.90, 1.16)	1.31 (1.01, 1.69)	0.13	1.02 (0.88, 1.19)	1.14 (0.97, 1.36)	0.49	0.91 (0.69, 1.19)	1.37 (1.17, 1.60)	0.003*
0–60 days before event	0.99 (0.90, 1.09)	1.28 (1.04, 1.56)	0.17	1.02 (0.90, 1.14)	1.08 (0.94, 1.23)	0.71	0.87 (0.69, 1.08)	1.33 (1.18, 1.50)	0.0002*
0–90 days before event	1.03 (0.94, 1.12)	1.25 (1.04, 1.49)	0.67	1.06 (0.96, 1.18)	1.07 (0.95, 1.20)	0.75	0.91 (0.75, 1.09)	1.34 (1.21, 1.49)	< 0.01*
0–180 days before event	1.19 (1.11, 1.28)	1.31 (1.13, 1.51)	0.90	1.18 (1.09, 1.29)	1.25 (1.14, 1.37)	0.91	1.10 (0.94, 1.28)	1.44 (1.32, 1.57)	0.001*
0–365 days before event	1.46 (1.37, 1.55)	1.587 (1.41, 1.79)	0.56	1.43 (1.33, 1.54)	1.55 (1.43, 1.68)	0.74	1.29 (1.13, 1.46)	1.69 (1.58, 1.82)	0.002*

*Indicates Statistical Significance at *p* < 0.05.

## Discussion

Our study found that ILI is a significant risk factor for acute cardiovascular events, including stroke and MI. There is a 39% increased odds of stroke and 24% increased odds of MI during the first 15 days after ILI, however, this risk drops after 15 days for MI while it remains elevated for stroke. The odds of stroke or MI 1 year after ILI remains elevated, however, the risk was stronger for stroke than MI (69 and 48% increased odds, respectively). This was predominantly driven by the risk in the first 15 days for MI. The timing of stroke and MI after ILI are consistent with prior reports that have highlighted how the risk for MI is higher shortly after ILI, while the risk of stroke remains increased for up to a year [7, 15].

To our knowledge, this is the first study to investigate whether the relationship between ILI and stroke, or ILI and MI, differs by sex, race-ethnicity, or by urban-rural status. We found the relationship between ILI and stroke was overall stronger for men and people living in rural areas, but differences were marked in the 90 days prior to stroke event. We found no difference in the relationship between ILI and stroke by race/ethnicity. The overall risk of stroke is higher in men and people living in rural areas which could explain some of the increased risk of stroke after ILI in these groups. However, the relationship between ILI and stroke did not differ between racial groups, despite stroke risk differing by race-ethnicity.

This was opposite to the findings for MI, where the relationship between ILI and MI was strongest in White patients compared to Black patients in the year prior to the event. We saw no difference in the ILI-MI relationship by sex or urban/rural status. These findings could be a reflection of differences in factors such as socioeconomic status and decreased access to health systems, as opposed to biological mechanisms [28]. It could be possible that people with stroke risk factors have more access to care- therefore adding to a higher probability of being vaccinated. Additional study is needed to evaluate whether the relationship between ILI and stroke/MI is different in people with diabetes or chronic heart failure, and whether there are additional patient characteristics that are associated with greater risk of stroke or MI after ILI. Additionally, these findings could be a reflection of regional influenza vaccine usage in NY State, as vaccination for influenza has been shown to reduce the risk of stroke and MI after ILI [10]. Women, people living in urban areas, and people with cardiovascular risk factors are more likely to be vaccinated for influenza [10]. Despite this, influenza vaccination in adults in NY state remains below the Centers for Disease Control goal of 90% with a range of 33–67% vaccinated depending on age group, with vaccinations increasing with age.

Proposed mechanisms behind the relationship between ILI and stroke or MI include mediation through a pro-thrombotic state, inflammation-mediated injury of endothelium, or through effects on cardiac endothelium [6, 10–13]. The relationship between the innate immune system and stroke risk has been long described. While the mechanism of stroke risk remains poorly understood, several hypotheses implicate an increased immune response as a potential mechanism for the increased stroke risk. Additional explanations include polymorphisms in the toll-like receptor 4 gene (TLR4), involved in the innate immune system’s response to both bacterial pathogens and influenza [29–31].

The increasing recognition of the importance of inflammatory pathways in stroke and cardiovascular disease is consistent with a possible role of peptide sharing-based cross-reactivity as contributing factors to cerebrovascular damage [32]. The results of this study suggest the population at risk for MI post-infection is different than the population at risk for stroke post-infection due to the differences in the risk periods. While overlap exists between the two populations in terms of risk, the two outcomes need to be assessed separately to distinguish the most at-risk populations, to target potential interventions for future studies to the appropriate at-risk groups. The degree of stroke risk can vary significantly from person to person, however, the use of the case-crossover study is a strength in this regard. More research should be performed on the risk of stroke and MI after ILI to determine which preventative measures are most appropriate for reducing stroke and MI risk after ILI. Future studies are needed to evaluate whether the differences in risk for stroke versus MI after ILI by subgroups provide insight into potential differences in mechanisms, including social, behavioral, or biologic mechanisms.

There were several limitations to our study. The use of an administrative dataset limits us to defining the outcomes, exposures, and covariates based on ICD-9 codes. While these ICD-9 codes have been validated, there was the potential for misclassification. For example, we did not have information on severity of illness, or on outpatient visits or treatments. A key limitation of this study is that we are only able to identify ILI cases severe enough to require treatment in the ED or inpatient settings and likely biasing the effect of ILI on stroke and MI towards the null. Additionally, we did not have any information on influenza vaccination. We did not expect any outcome misclassification to be associated with ILI and any resulting bias, therefore, would bias our results toward the null. The case-crossover design also does not account for the increased risk associated with the aging of the patient over time, their concomitant development of new risk factors (e.g. newly diagnosed diabetes etc.), or cases of ILI or other infection that did not warrant a visit to the ED. To attempt to address this, we limited the control periods to 2 years. Despite these limitations, the use of the NY State DOH SPARCS data provides information on nearly all in-hospital admissions regardless of insurance status resulting in a large, generalizable study. The case-crossover design reduces the influence of confounders on the measures of association. The ability to explore group differences in the relationship between ILI, stroke and MI identified at risk groups who could be targeted for future interventions in decreasing the risk of stroke or MI after ILI.

## Conclusions

Our study highlights risk period differences for acute cardiovascular events after ILI, potentially indicating differences in mechanism behind the risk of stroke after ILI compared to the risk of MI after ILI. We found that high risk populations for stroke after ILI include men and people living in rural areas, while whites are at high risk for MI after ILI. Future studies building on this data may help identify targeted interventions to mitigate these risks.

## Data Availability

The data that support the findings of this study are available from the New York State Department of Health and are available by written request and after research proposal is approved. Restrictions apply to the availability of these data, which were used under license for the current study, and are not publicly available. Data are available from the authors upon reasonable request and after obtaining permission from the New York State Department of Health.

## Abbreviations

MI: Myocardial infarction
ILI: Influenza-like-illness
SPARCS: New York statewide planning and research cooperative system
OR: Odds ratio
CI: Confidence intervals
CVD: Cardiovascular disease
ED: Emergency department
ICD-9: International classification of disease
RUCA codes: Rural-urban commuting area codes
